# Modification of Kraft Lignin with Dodecyl Glycidyl Ether

**DOI:** 10.1002/open.201900263

**Published:** 2019-10-10

**Authors:** Norah S. Alwadani, Pedram Fatehi

**Affiliations:** ^1^ Chemical Engineering Department Lakehead University Thunder Bay ON Canada P7B5E1

**Keywords:** lignin, thermal analysis, biorefining, NMR spectroscopy, methylation reactions

## Abstract

Kraft lignin (KL) is extensively produced in industry but is mainly burned as fuel. To broaden its use, KL was grafted with dodecyl glycidyl ether to alter its thermal properties. The reaction of KL with dodecyl glycidyl ether (DGE) was analyzed using nuclear magnetic resonance (NMR), Fourier infrared spectroscopy (FT‐IR) and elemental analysis. Alternatively, KL was methylated to mask its phenolic hydroxy groups to investigate how phenolic hydroxy groups impact the grafting of the alkyl chain of DGE onto lignin (methylated Kraft lignin, MKL). The methylation facilitated the molecular weight enhancement and thermal stability reduction of Kraft lignin via grafting with DGE. The influence of grafting alkyl chains on the structural and thermal properties of KL and MKL was studied using thermogravimetric analysis and differential scanning calorimetry analysis. Our data suggest that, due to their high molecular weights and lower glass transition temperatures, the produced lignin derivatives may be promising feedstocks for composite production.

## 1 Introduction

Today, the production of chemicals and polymers relies largely on the use of fossil‐based resources, which are becoming increasingly scarce and expensive. As such, the chemical industry is currently seeking sustainable processes that utilize renewable resources. Recently, the replacement of fossil‐based products with biomass‐based products has been actively investigated.^1^, ^2^ In particular, lignocellulosic materials are considered to be promising feedstocks since they are plentiful, inexpensive and renewable.^1^, ^3^, ^4^, ^5^, ^6^, ^7^

Lignin is one of the main components of biomass.^8^, ^9^, ^10^, ^11^, ^12^ Lignin has primarily been used as a fuel source, but recent studies have shown that it could be converted into several value‐added products.^5^, ^13^, ^14^, ^15^ Using lignin as a feedstock for producing value‐added products has some advantages and disadvantages. Lignin is not used as food, but it is an underutilized by‐product of many food and pulping processes.

Additionally, there are well‐established commercial processes for producing different forms of lignin in a wide range of qualities and quantities.^16^, ^17^ However, the downside is that lignin is a chemically complex material, which makes its utilization challenging.^18^, ^19^, ^20^ Although many products are profitably produced from cellulose, the generation of lignin‐based products has not been commercialized yet.

Numerous studies have been conducted over the past few years to widen lignin's applications.^21^, ^22^, ^23^, ^24^, ^25^, ^26^, ^27^ Many studies have reported different routes and ideas for lignin modification to alter its physical and thermal properties.^22^, ^23^ Esterification and etherification have been proposed to change the physical (solvophobicity) and thermal (glass transition temperature) properties of lignin.^22^, ^23^, ^24^ In one study, soda lignin was propylated in an alkaline environment for its potential use in polyurethane foam formulations.^25^ Gordobil and co‐workers^26^ have esterified organosolv lignin with dodecanoyl chloride in N,N‐dimethylformamide (DMF). The resulting product showed excellent performance as a coating barrier in wood product manufacturing because of its low glass transition temperature.^26^ Similarly, the alkoxylation of lignin was claimed to improve lignin's compatibility in polyurethane and polymer blend products.^27^

Thermal degradation temperature, glass transition temperature, and ignition temperature are crucial indicators for a material's thermal stability, which may affect a product's use in composites.^28^, ^29^ Therefore, the thermal behavior of lignin derivatives should be investigated to widen their application in, for example, composites. Researchers have performed different reactions to alter lignin's properties by lowering the glass transition temperature and increasing lignin‘s thermoplasticity.^21^, ^26^ In this work, we examined the etherification of KL as a means to change its thermal behavior.

Methylation has been used in the past to improve the properties of lignin for use in composites.^30^ One study used dimethyl carbonate (DMC) to methylate KL and reported that this reduced the glass transition temperature (T_g_) of the KL. Since methylation restricts the intermolecular hydrogen bonding of the phenolic‐OH groups, the decrease in T_g_ was related to the reduction in the free hydroxy groups of KL as a result of methylation.^27^ In another study, the authors investigated the methylation selectivity of softwood KL using methyl iodide and dimethyl sulfate and reported that the selectivity and efficiency of dimethyl sulfate were greater than those of methyl iodide.^31^ Methylation masks the phenolic‐OH groups of lignin; hence, it may be used to facilitate analysis on the mechanism of the etherification reaction of lignin.^31^, ^32^ Also, methylation promotes the thermal response of KL since the intramolecular hydrogen bonds are greatly reduced after complete derivatization. Therefore, methylation may improve the performance of the etherification reaction and the properties of resulting etherified Kraft lignin‐based products.^33^ Therefore, one objective of our work was to study the thermal stability of lignin after methylation and chain extension via etherification.

The main novelties of this work are the analysis on 1) the reaction mechanism of KL and dodecyl glycidyl ether (DGE) with DGE as the etherifying agent, 2) the impact of methylation on the etherification of KL, and 3) the thermal behavior of the induced lignin derivatives. KL was chosen for this analysis as it is commercially available and has a higher number of phenolic‐OH groups compared to other types of lignin.^3^

## 2 Results and Discussion

### 2.1 Dodecyl Glycidyl Ether (DGE) Characterization

Various glycidyl ethers have been produced via different routes as the starting materials for synthesizing alcohols.^34^, ^35^, ^36^ Alcohols can be reacted with epichlorohydrin to generate glycidyl ethers.^37^ In this work, dodecyl glycidyl ether (DGE) was prepared according to the method reported by Chen and co‐workers.^3^

**Scheme 1:**
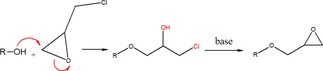
The reaction route of epichlorohydrin and 1‐dodecanol for glycidyl ether production.

Scheme 1 shows the reaction pathway for preparing glycidyl ethers. Epichlorohydrin was the source of the epoxide ring and 1‐dodecanol with a 12‐carbon aliphatic chain was chosen to attach to the epoxide ring via an ether bond. 1‐dodecanol was chosen because it has a single hydroxy group. Using a corresponding di or polyalcohol would extend the degree of reaction, generating products with undesired properties, e. g., uncontrollable viscosity, which could not be used in lignin modification.^36^ The reaction was carried out in steps. First, the alcohol was deprotonated in the presence of a base (NaOH), then the catalyst (quaternary ammonium salt) transferred the charged derivative to the liquid where the reaction with epichlorohydrin took place.

The experimental results confirmed that DGE had an epoxide equivalent weight (EEW) of 212.63 g/mol. Comparing this to the theoretical value (242.4 g/mol), it can be stated that the product contained a small amount of impurities, which contributed to the smaller experimental EEW. Additionally, some diol may have been formed as the product of side‐reactions. ^1^H NMR was used to determine the ratio of components in the product mixture (DGE and diol). The ^1^H NMR spectrum of the DGE product is shown in Figure S1 (in the supplementary materials), and the inset shows a zoomed‐in view of the resonance signal of the epoxide group. The resonance signal of the epoxide group was reported to be between 2.5 ppm to 4.5 ppm.^38^, ^39^, ^40^, ^41^ Therefore, based on ^1^H NMR results, 50 % of the epoxide rings in DGE were opened during the reaction, lowering the yield of the reaction to an average of 40 % after washing and purification.

### 2.2 Methylation Performance

In this study, dimethyl sulfate was chosen as the source of methyl groups to mask the phenolic hydroxy groups of lignin. The methylation of KL took place under the reaction conditions described in the literature.^27^, ^31^, ^42^ Scheme S1 shows the methylation reaction of lignin, while Figure S2 shows the ^31^P NMR spectra of KL and MKL in pyridine/CDCl_3_ mixture (1.6/1 v/v). On the ^31^P‐NMR spectra, the peaks in the range of 150.0–145.5 ppm can be attributed to aliphatic‐OH groups, while those in the range of 144.5–137.0 ppm can be attributed to phenolic‐OH groups.^42^

The areas under the peaks in Figure S2 were used to quantify the functional groups attached to the lignin samples, and the results are tabulated in Table 1. As a result of methylation, condensed and non‐condensed phenolic‐OH group contents significantly decreased from 0.47 mmol/g and 0.68 mmol/g to 0.06 mmol/g and 0.08 mmol/g, respectively. Condensed phenolic‐OH groups are defined as those that belong to aromatic rings and have a substituent in the 5^th^ carbon position of the aromatic ring. Non‐condensed phenolic‐OH groups have no such substituents. It was reported that the reaction rate of the non‐condensed phenolic‐OH groups of lignin was faster than that of the condensed ones.^31^, ^32^ The low reactivity of the condensed phenolic hydroxy groups is due to the sterically hindered environment of these groups.^32^ The electron‐donating effect of ether or methylene groups (through the mesomeric effect or positive inductive effect) likely increased the nucleophilicity of the phenoxide ions. Moreover, the existence of these groups (ether or methylene) on neighbouring carbons may have provided the rotational freedom that increased the groups’ reactivity and accessibility to condensed phenolic‐OH groups.^27^ Therefore, the combination of sterically and electronically favourable conditions likely made non‐condensed phenolic‐OH groups more reactive.^32^, ^42^

**Table 1:** The hydroxy groups content analysis of KL and MKL conducted by an automatic potentiometric titrator and ^31^P NMR.

^31^P NMR					Titration	
Groups	KL		MKL		KL	MKL
	mmol/g	%	mmol/g	%	mmol/g	mmol/g
Aliphatic	0.21	14.1	1.36	83.8	‐	‐
Condensed	0.47	31.7	0.06	3.5	‐	‐
Non‐condensed	0.68	45.9	0.08	5.2	‐	‐
Carboxylic‐OH	0.12	8.1	0.12	7.5	‐	‐
Phenolic‐OH*					1.2±0.1	0.12±0.1
Aliphatic‐OH*					0.33±0.1	0.33±0.12

* Data is shown as the average of 5 independent experiments +/‐ standard deviations (SD).

Both titration and NMR analyses confirmed that phenolic‐OH content was significantly decreased after methylation, which would imply the successful conversion of phenolic‐OH groups to methoxy (OCH_3_). The remaining phenolic‐OH groups were probably not accessible to dimethyl sulfite for the methylation reaction (e. g., via steric hindrance). As NMR analysis is proportionality‐based, the decrease in phenolic‐OH group content led to an increase in aliphatic‐OH group content (Table 1). In titration analysis, the aliphatic‐OH groups were not affected by the methylation reaction, which confirmed that the selectivity of methylation to phenolic‐OH groups was high. Quantitative ^31^P NMR data showed that about 87.5 % of the phenolic hydroxy groups were converted to methoxy groups in MKL production (Table 1), indicating that the phenolic‐OH groups were successfully converted to methoxy groups.

Table 2 lists the organic contents of KL and MKL (on an ash‐free basis). The results show that methylation increased carbon and hydrogen contents, while it decreased oxygen and sulfur contents. These changes can be attributed to the conversion of hydroxy groups to methoxy ones, while impurities in the KL, such as salts and ashes, likely caused the minor reductions in oxygen and sulfur contents.

**Table 2:** The elemental composition and molar mass of KL and MKL.

Sample	Elemental analysis^*^ (wt.%)				MW^*^ (g/mol)
	C	H	O^a^	S	
KL	63.75	5.81	29.17	1.26	(1.13± 0.15)×10^6^
MKL	65.94	5.99	26.94	1.13	(3.09± 0.16)×10^6^

a: by difference, * Data is shown as the mean of 3 independent experiments +/−SD.

### 2.3 Reaction of Kraft Lignin Derivatives and DGE

**Scheme 2:**
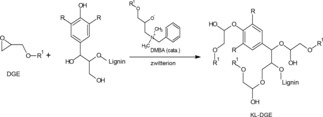
Reaction of lignin and DGE in the presence of DMBA, where R=OCH_3_ or H, R^1^=CH_2_−(CH_2_)_10_−CH_3_.^19^

Six samples were prepared with different molar ratios of DGE to lignin hydroxy group content in DMSO at 100 °C for 5 h with both KL and MKL. Typically, the reaction between an epoxide ring and the hydroxy groups in KL molecules can be accelerated by the addition of Lewis bases.^43^ Given the low reactivity of KL, dimethylbenzylamine was used as the catalyst for DGE incorporation. The reaction of KL and DGE is shown in Scheme 2. The epoxide ring reacts with the catalyst (tertiary amine) to form a zwitterion (due to its high basicity), which then reacts with the hydroxy groups of lignin. The molar ratio of DGE/lignin was 0.348, 0.466 and 0.937 to produce KL‐1, KL‐2 and KL‐3, respectively, with a yield of approximately 75 %. The same ratios were used to produce MKL‐1, MKL‐2, and MKL‐3 with a yield of approximately 80 %.

### 2.4 Structural Analysis of KL‐DGE Product

**Figure 1:**
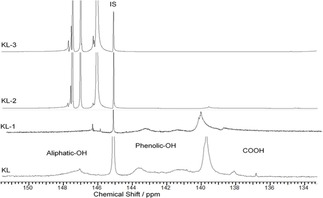
^31^P‐NMR spectra of KL, KL‐1, KL‐2, and KL‐3 in pyridine/CDCl_3_ mixture (1.6/1 v/v), IS: internal standard.

**Figure 2:**
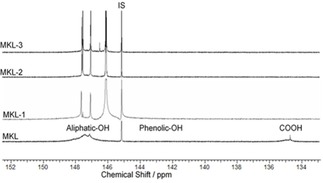
^31^P‐NMR spectra of MKL, MKL‐1, MKL‐2 and MKL‐3 in pyridine/CDCl_3_ mixture (1.6/1 v/v), IS: internal standard.

The products of the KL and DGE reactions were characterized by ^31^P‐NMR in Figure 1 for KL, KL‐1, KL‐2, and KL‐3. The ^31^P NMR spectra of MKL samples are available in Figure 2.

According to the literature,^44^ the assigned peaks for aliphatic‐OH, phenolic‐OH, and carboxylic‐OH groups in KL are in the range of 150.0–145.4 ppm, 144.5–137.0 ppm, and 136.0–134.0 ppm, respectively. Indeed, three new peaks in the aliphatic‐OH range were noticed (147.9–147.4, 147.1–146.8, and 146.6–145.8 ppm) in the spectra of KL‐1, KL‐2, and KL‐3, the magnitude of which increased as the DGE/lignin molar ratio increased. Overall, these results confirmed that the grafting of DGE to lignin was proportional to the amount of DGE present in the reaction.

Based on the ^31^P‐NMR analysis, the hydroxy group content of the lignin samples was determined, and the results are shown in Table 3. Carboxylic‐OH and phenolic‐OH group contents dramatically decreased, while aliphatic‐OH group content increased as more DGE was grafted to lignin. The decrease in carboxylic‐OH group content may explain the different charge densities and water solubilities of the lignin derivatives. However, the total OH content of lignin derivatives was unchanged, suggesting that hydroxy groups were converted from one type to another. NMR analysis shows that the degree of substitution increased as DGE content increased.

**Table 3:** Quantification of hydroxy groups of KL and MKL before and after reaction with DGE using ^31^P‐NMR.

Samples	Hydroxy content^*^ (mmol/g of KL)				DS_NMR_^*^
	Phenolic‐OH	Aliphatic‐OH	Carboxylic‐OH	Total OH	
KL	1.15	0.21	0.12	1.48	–
KL‐1	1.13	0.35	ND	1.48	0.22
KL‐2	0.08	1.41	ND	1.49	1.90
KL‐3	ND	1.48	ND	1.48	2.01
MKL	ND	1.36	0.12	1.48	–
MK‐1	ND	1.47	ND	1.47	0.07
MKL‐2	ND	1.48	ND	1.48	0.56
MKL‐3	ND	1.48	ND	1.48	1.13

ND: not detected, * Data is shown based on the average of 3 independent experiments as the weight gain was considered in the calculations.

The properties of modified KLs and MKLs are listed in Table 4. Carbon and hydrogen contents increased, while oxygen and sulfur contents decreased as the ratio of DGE/lignin increased. This is due to the corresponding increase in DGE attachment to KL or MKL.

**Table 4:** The characteristics of KL‐DGE and MKL‐DGE products (based on ash free analysis)

Sample	Elemental analysis^*^ (wt. %)				MW^*^ (g/mol)
	C	H	O^a^	S	
KL	63.75	5.81	29.17	1.26	(1.1± 0.1)×10^6^
KL‐1	66.71	6.25	24.90	2.14	(5.5± 3.6)×10^6^
KL‐2	67.28	7.18	24.59	0.93	(1.8± 2.1)×10^7^
KL‐3	67.32	7.27	24.54	0.86	(4.1± 0.3)×10^7^
MKL	65.94	5.99	26.94	1.13	(2.36± 0.16)×10^6^
MKL‐1	67.43	6.86	25.10	0.60	(3.09± 0.47)×10^6^
MKL‐2	68.04	6.95	24.24	0.76	(1.43± 0.84)×10^7^
MKL‐3	68.81	7.12	22.98	1.08	(4.50± 4.10)×10^7^

a: by difference, based on ash‐free analysis, * Data is shown in the mean of 3 independent experiments +/‐ SD. b: based on ash‐free formula

Similarly to the ^31^P‐NMR analysis, the elemental analysis showed a small difference between the chemical compositions of KL‐2 and KL‐3. MKL derivatives (MKL‐2 and MKL‐3) showed a slight increase in the carbon and hydrogen contents as well. The grafting ratio increased as the molar ratio of DGE/KL and DGE/MKL increased, as shown in Table 4.

The molecular weight of KL‐1, KL‐2 and KL‐3 increased as the ratio of DGE/KL increased (Table 4). The general trend supports the fact that grafting DGE to KL increased the molecular weight of lignin. At a lower DGE/KL ratio, DGE might only attach to the carboxylic‐OH group, given that the reaction between the carboxylic‐OH and glycidyl ether groups occurred first due to the relatively high acidity of carboxylic‐OH groups in lignin.^45^ However, at a higher DGE/KL ratio, DGE was also grafted to lignin at the phenolic‐OH reactive site, leading to a reduction in phenolic‐OH group content and an increase in molecular weight. Based on the reaction mechanism, the following phenomenon can be hypothesized: carboxylic‐OH groups react preferentially (compared to phenolic‐OH) with DGE because of their relatively high acidity. This was clear in the ^31^P‐NMR spectrum; the peak in the range of 136.0–134.0 ppm, which corresponded to carboxylic‐OH groups, did not appear while the aliphatic‐OH peak increased slightly (Figure 1).

Moreover, ^31^P‐NMR results showed that increasing the ratio of DGE/lignin decreased the aromatic‐OH content of lignin (Figure 1). These results provide proof of DGE grafting at the phenolic‐OH position, as phenolic‐OH groups are more reactive than aliphatic‐OH in lignin moieties.

**Figure 3:**
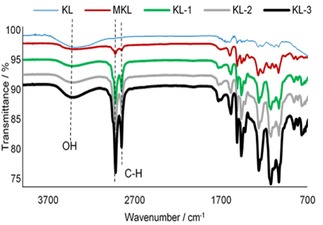
FT‐IR spectra of KL, MKL, KL‐1, KL‐2, and KL‐3.

The FT‐IR spectra of KL, KL‐1, KL‐2, and KL‐3, as well as MKL, are shown in Figure 3. According to the literature,^46^, ^47^ the stretching bond between 3400 and 3100 cm^−1^ corresponds to the O−H bond of aliphatic hydroxy and phenolic hydroxy groups, while the absorption peaks at 2930 cm^−1^ and 2300 cm^−1^ correspond to stretching of C−H and C=C bonds in aldehyde groups.^46^, ^47^ A comparison between the KL samples indicates that there is a noticeable decrease in the stretching bond between 3400 and 3100 cm^−1^, which may be due to reduced carboxylic‐OH or phenolic‐OH contents of KL after DGE attachment. The slight increase in the intensity of absorption peaks at 2924 and 2853 cm^−1^ is likely due to the introduction of CH_3_ groups to KL by methylation, while a significant increase was noticed at the same peaks via the DGE reaction, which indicates that the long alkyl chain (DGE) was successfully introduced to KL.

### 2.5 Degree of Substitution and Molecular Weight Analyses of MKL‐DGE Products

The degree of substitution (DS) in MKL increased as the molar ratio of DGE/lignin increased from 0.07 to 1.13 (Table 3). However, as the methylated samples had fewer reactive sites than KL, the DS was generally lower for the MKL samples than for the KL ones.

Interestingly, the DS_NMR_ values for the methylated lignin samples were lower than those for the unmodified lignin samples. This corroborates the results shown in Figure 1 and Table 1 pertaining to the reduction in phenolic‐OH group content. Furthermore, the molecular weight of the MKL samples increased as the DGE/lignin ratio increased. These molecular weights were larger than those reported for modified KL due to the presence of the methyl group (rather than the hydroxy group) on the phenyl group of lignin (Table 3).

### 2.6 Thermogravimetric Analysis (TGA)

**Figure 4:**
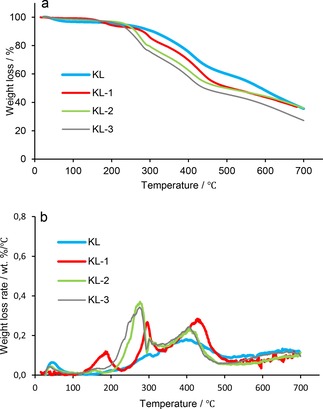
a) Weight loss and b) weight loss rate of KL, KL‐1, KL‐2 and KL‐3 conducted under N_2_ at a flow rate of 30 mL/min with a heating rate of 10 °C/min.

**Figure 5:**
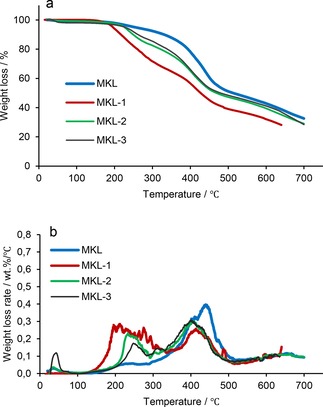
a) Weight loss and b) weight loss rate of MKL, MKl‐1, MKL‐2 and MKL‐3 conducted under N_2_ at a flow rate of 30 mL/min heated at 10 °C/min.

Figures 4 and 5 show the TGA spectra of the lignin samples. Compared to KL, MKL samples exhibited lower thermal stability. Interestingly, the modified lignin samples displayed a similar thermal degradation pattern irrespective of their methylation pre‐treatment. DGE‐modified KL samples exhibited lower thermal resistance than unmodified lignin, and this decrease in the stability of modified lignin is attributed to the decomposition of alkyl chains.^48^

The weight loss below 100 °C is attributed to the elimination of moisture in all samples. The lignin samples started to decompose at a temperature higher than 200 °C. Brebu and co‐workers also reported that the lignin degradation started between 230 °C and 260 °C, which was attributed to the decomposition of the propanoid side chain in lignin.^48^ It is observed that KL‐1 had a decomposition peak below 200 °C (Figure 4), however, by increasing the grafting ratio, the degradation temperature rose, due to the fact that the replacement of hydroxy groups with DGE reduced the amount of hydrogen bonding in the lignin molecule in the first decomposition step.^49^ According to the literature, the weight loss rate of lignin between 440 and 500 °C was ascribed to the breakdown of intermolecular bonding within lignin.^50^, ^51^ Furthermore, MKL samples generally had greater thermal stability, which is due to masking of phenolic‐OH groups by the methylation reaction (Figure 5).

Table 5 lists the thermal degradation temperatures of lignin samples derived from TGA analysis. The temperature at which 10 % of weight loss occurred (T_10%_) was found to decrease significantly by increasing the ratio of DGE/lignin (T_10%_=307, 280, 260 and 246 °C for KL, KL‐1, KL‐2, and KL‐3, respectively). Similarly, 50 % of the mass loss occurred at markedly lower temperatures compared to unmodified KL. Furthermore, by increasing the molar ratio of DGE/lignin, the ash content of lignin samples dropped. The results for methylated samples followed similar trends, but generally, the methylated samples had lower thermal degradation temperatures. The decrease in the thermal stability of lignin due to methylation could be a disadvantage for its end‐use applications.^52^

**Table 5:** Thermal degradation temperatures at 10 % (T_10%_) and 50 % (T_50%_) weight loss of lignin samples, and an ash content of lignin samples left at 700 °C (R_700_).

Samples	T_10%_ (°C)	T_50%_ (°C)	T_ign_	Ash content
KL	307	589	300	35
KL‐1	280	510	280	36
KL‐2	260	498	242	35
KL‐3	246	441	239	27
MKL	319	512	341	32
MKL‐1	261	479	290	28
MKL‐2	249	468	286	29
MKL‐3	216	447	277	28

Moreover, the ignition temperatures (T_ign_) of the lignin samples were determined using TGA results. The ignition temperature of lignin‐based products is the line tangent to two different temperatures, at which the weight loss of lignin starts and the maximum weight loss rate of lignin occurs.^53^ By increasing the grafting ratio of DGE/lignin, T_ign_ decreased due to the increase in carbon content and decrease in ash content (Table 5). The results in Table 5 also depict that 1) MKL derivatives had less ash than KL derivatives and 2) DGE grafting slightly reduced the ash content of lignin. As such, it seems that the reaction environment (either methylation or grafting) facilitated the removal of inorganic ash from KL during the modification process. This phenomenon was also seen in the past.^54^

### 2.7 Differential Scanning Calorimetry (DSC) Analysis

To investigate the changes in the thermal behavior of KL derivatives, the glass transition temperature, T_g_, of the samples was determined, and the results are presented in Table 6. As reported previously,^49^, ^55^ the T_g_ of lignin varies depending on the type of lignin and the process through which the lignin is produced. The T_g_ of KL is usually in the range of 90 and 170 °C. As shown in Table 6, the T_g_ of KL was found to be 154 °C. Interestingly, grafting DGE to KL reduced the T_g_ values to 117 °C, 89 °C and 70 °C for KL‐1, KL‐2 and KL‐3, respectively. The T_g_ of lignin is affected by its molecular weight, crosslinking structure, and hydrogen bonds.^29^, ^56^ Grafting DGE to lignin reduces the number of available hydroxy groups that can partake in hydrogen bonding.^27^ As more DGE was attached to KL, its structure became less condensed and its entanglement ability with the aliphatic chain was reduced, which caused a reduction in T_g_. The MKL had a lower T_g_ (101 °C) compared to KL due to its lower hydroxy group content and, therefore, lower hydrogen bonding capacity. The T_g_ values of MKL derivatives were found to be 69 °C, 68 °C, and 67 °C for MKL‐1, MKL‐2, and MKL‐3, respectively.^27^

**Table 6:** Thermal properties of KL derivatives measured by DSC.

Lignins	T_g_ (°C)	Cp (J/g.°C)
KL	154.33	0.2920
KL‐1	116.82	0.2882
KL‐2	89.23	0.2736
KL‐3	70.19	0.2573
MKL	101.19	0.3386
MKL‐1	69.46	0.2961
MKL‐2	68.53	0.2741
MKL‐3	67.57	0.2626

According to the literature, T_g_ depends on the molecular weight, the concentration of entanglements, and interaction forces between highly polar groups in a polymer matrix.^57^ However, the chain entanglements may not be critical in hyperbranched polymers with condensed structures. Therefore, it may not be possible to obtain a direct correlation between T_g_ and molecular weight for hyperbranched polymers. In other words, different T_g_ values can be obtained for hyperbranched polymers of similar molecular weights by changing their core functional groups.^57^ An increase in end‐group free volume decreases a polymer's T_g,_ as end‐group free volume is often a prevailing factor governing T_g_.^57^, ^58^ In this study, the reduction in T_g_ could also be attributed to increasing the grafting ratio of end‐group free volumes (i. e., DGE). As more free volume became available to the end units of lignin, molecular mobility increased and T_g_ decreased.^58^

The heat capacities at constant pressure (C_p_) for the lignin samples are listed in Table 6. Increasing the ratio of DGE/lignin caused the C_p_ values to decrease, as this increased the carbon and hydrogen contents of the lignin‐based products.^51^ Therefore, the heat capacity of the lignin samples decreased as the free volume in the molecules increased due to the reduction in hydrogen bonding capacity.^59^, ^60^, ^61^ This behavior is attributed to the enhancement in the free volume of the KL derivatives, which led to a corresponding increase in chain mobility.^21^, ^62^ As MKL had a higher molecular weight (Table 2), its C_p_ was higher than that of KL. These results suggest that crosslinked KL molecules were not as dense as methylated ones, as the latter had higher C_p_ values.^62^ However, the production of KL derivatives via attaching long aliphatic chains appears to be an appropriate method to obtain lignin‐based products with suitable glass transition temperatures (T_g_) for use in, for example, composites and polyols for polyurethane applications.^25^, ^29^

## 3 Conclusions

The present work successfully demonstrated the grafting of long‐chain dodecyl glycidyl ether (DGE) to KL in the presence of N,N‐dimethylbenzylamine as a catalyst. KL was pretreated with dimethyl sulfate to mask the phenolic hydroxy groups. ^31^P NMR and titration confirmed the conversion of hydroxy groups to methoxy groups as the total condensed and non‐condensed phenolic‐OH groups of lignin were significantly decreased by methylation from 1.15 to 0.14 mmol/g, and from 1.20 to 0.12 mmol/g, respectively. Moreover, the aliphatic hydroxy groups were unaffected by methylation. ^31^P NMR also confirmed the grafting ratio of DGE to unmodified and modified KL. Interestingly, DGE was grafted at the carboxylic‐OH group first due to this group's higher acidity. By increasing the DGE/lignin ratio in the reaction, the phenolic‐OH groups also reacted with DGE. The results on the MKL were similar to those of untreated KL, but lower grafting ratios were obtained, as the phenolic‐OH groups of methylated lignin were masked.

The thermal stability of lignin was reduced by increasing the molar ratio of DGE/lignin. Alkoxylation with DGE impaired the thermal stability of lignin by increasing the crosslinking and entanglement of molecules. Interestingly, MKL derivatives showed less thermal stability than unmodified ones, which was due to the decrease in hydrogen bonding of lignin via methylation. Methylation decreased the glass transition temperature of KL. The glass transition temperature was further decreased by grafting lignin with DGE. Therefore, MKL derivatives showed lower glass transition temperatures than KL derivatives.

## Experimental Section

### Materials

Softwood KL was supplied by FPInnovations from its pilot plant facilities located in Thunder Bay, ON. Canada. 1‐dodecanol (98 %), epichlorohydrin (ECH), tetrabutyl ammonium bromide (TBAB), sodium hydroxide (98 %), toluene, N,N‐dimethylbenzylamine (BDMA), petroleum ether, hydrochloric acid (37 %), dimethyl sulfoxide (DMSO), dimethyl sulfate, pyridine, deuterated chloroform (CDCl_3_, 99.8 %), cyclohexanol, chromium (III) acetylacetonate,2‐chloro‐4,4,5,5‐tetramethyl‐1,2,3 dioxaphospholane (TMDP, 95 %), N,N‐dimethylformamide (DMF, 99.8 %), poly(diallyldimethylammonium chloride) solution (PDADMAC; 100,000‐200,000 g/mol, 20 wt.% in water), anionic polyvinyl sulfate (PVSK; 100,000‐200,000 g/mol, 98.4 wt.% esterified), potassium hydroxide solution (8 M), para‐hydroxybenzoic acid, methanol and phenolphthalein, all analytical grades, were purchased from Sigma Aldrich and used as received. Dialysis membrane (molecular weight cut‐off of 1000 g/mol) was obtained from Spectrum Labs Inc., USA.

### Reaction Procedure

Figure S3 shows the reaction procedures followed to produce various modified lignin samples: 1) etherifying agent (dodecyl glycidyl ether, DGE) was prepared; 2) MKL was also prepared via the methylation reaction of KL and 3) DGE was grafted on KL and MKL.

### Dodecyl Glycidyl Ether (DGE) Preparation

The DGE was prepared according to the method described by Chen et al.^37^ 1‐dodecanol was dissolved in toluene (0.99 mol) and combined with 25 g of 48 wt.% NaOH (aq) and 1.6 g of TBAB in a 250‐mL round‐bottom flask. Then, 18.5 g of ECH were added dropwise to the mixture at room temperature and stirred at 300 rpm. The mixture was heated at 50 °C for 6 h. Afterward, it was quickly cooled by immersing the flask in water, whereupon it was separated into an organic layer, which contained DGE, and an aqueous layer, which contained unreacted materials. The organic layer was separated and purified by washing with distilled water at 60 °C in a separatory funnel to extract unreacted materials, if any. Then, the solvent was completely removed from the organic layer using a rotary evaporator, Rotaryevap Buchi R210, at room temperature under vacuum to obtain DGE. The product was characterized comprehensively.

### Methylation of Kraft Lignin

To investigate the reaction mechanism of lignin with DGE, KL was methylated according to the procedure explained in the past.^31^, ^32^, ^42^ In this experiment, 1 g of KL was solubilized at room temperature in 15 mL of 0.7 M NaOH via stirring at 300 rpm. Dimethyl sulfate was added to the mixture at a 1/2.5 molar ratio of the phenolic‐OH group of lignin to dimethyl sulfate. At ambient temperature, the mixture was stirred for 30 min and then it was heated to 80 °C to react for 2 h. The pH of the mixture was maintained at 11–11.5 by continuously adding NaOH (0.7 M) to the mixture throughout the experiment. The pH of the reaction was kept at 11.5 by continuously adding NaOH to the reaction to compensate for the rapid hydrolysis of dimethyl sulfate that produced sulfuric acid. The phenolic‐OH group must be ionized to enable methylation, and this happens under alkaline pH.^31^ After the reaction, the mixture was cooled to room temperature, and then the product (MKL) was precipitated by decreasing the pH to 2 via adding 2 M HCl. Afterward, MKL was collected by filtration and thoroughly washed with deionized water until the sample pH reached neutrality. The MKL samples were then freeze‐dried (Labconco, FreeZone 1L).

### Lignin Modification with Dodecyl Glycidyl Ether (DGE)

KL and MKL were modified with DGE as described in the literature.^32^, ^45^ In a 250 mL round‐bottom three‐neck glass flask equipped with a mechanical stirrer, 2 g of lignin was dissolved under vigorous stirring (400 rpm) in 80 mL DMSO. Then, BDMA (0.45 g) was added as a catalyst, which was followed by DGE addition at different molar ratios relative to lignin's hydroxy group content. Afterward, the mixture was purged with nitrogen gas to remove oxygen. The first 30 min of the reaction occurred at room temperature, and then the mixture was heated to 100 °C and reacted for another 5 h. The reaction was stopped by adding 2 M HCl (5 mL), and the mixture was stirred for another 30 min at room temperature to cool down. Petroleum ether was then added to facilitate the separation of the products via centrifugation at 1500 rpm for 5 minutes. The solvent (DMSO) was then removed from the mixture via dialysis while changing water every hour for the first six hours, then every 6 h in the first day, and finally twice a day in the following day. The resulting water‐insoluble product was separated via centrifugation at 1500 rpm for 5 min, oven‐dried at 60 °C, and then labeled KL‐1, KL‐2, KL‐3 for modified KL and MKL‐1, MKL‐2 or MKL‐3 for modified MKL according to each sample's lignin/DGE ratio.

### Determination of the Epoxy Equivalent Weight (EEW) of Dodecyl Glycidyl Ether (DGE)

In this set of experiments, 2 g of DGE was added to 25 mL of 2 M pyridinium chloride (i. e., 1 mL of concentrated hydrochloric acid in 61.5 mL of pyridine). The mixture was then stirred and heated to 50 °C for 30 min. After cooling the mixture, a few drops of methanol (0.1 M) and phenolphthalein were added. The solutions containing DGE and a blank sample were titrated against 0.2 M NaOH. The EEW value of DGE in the solution was determined using equation (1), 63

(1) EEW=M/f(B-S)

where M is the mass (g) of DGE, f is the NaOH concentration (M), B is the amount of NaOH solution (mL) in the blank sample, and S is the amount of NaOH solution (mL) used for the dodecyl glycidyl ether sample.

### NMR Analysis

#### ^1^H‐NMR Analysis

The DGE sample was dissolved in CDCl_3_ at 20–30 mg/mL concentration. The solution was stirred for 30 min to fully dissolve the DGE. The ^1^H‐NMR spectrum was recorded at room temperature using an INOVA‐500 MHz instrument (Varian, USA) with a 45° pulse, 32 scans and interpulse delay time of 1.0 s.

#### ^31^P‐NMR Analysis

The hydroxy groups attached to KL were quantitatively analyzed by ^31^P‐NMR, which helped identify the success of the etherifying reaction.^31^, ^63^, ^64^, ^65^ The KL‐based samples (36.6 mg) were dissolved into 500 μL of pyridine/CDCl_3_ (1.6/1 v/v). Then, 35 μL of internal standard (21.5 mg/mL of cyclohexanol in 1.6/1 v/v pyridine/CDCl_3_) and 50 μL of T1‐relaxation agent (5.6 mg/mL of chromium (III) acetylacetonate in 1.6/1 v/v pyridine/CDCl_3_) were added to the mixtures and the mixtures were stirred at room temperature for 40 min. Afterward, 100 μL of TMDP was added as a phosphorylating agent for the detection and quantification of phenolic and aliphatic hydroxy moieties. The reaction mixtures were stirred at room temperature for another 10 min before transferring into a 5 mm NMR tube for NMR acquisition. The NMR spectra (in the range of 20 and 200 ppm) were recorded using an INOVA‐500 MHz spectrometer (Varian, USA) with a 90° pulse angle, 512 scans, and a 5 s interpulse delay.

### Degree of Substitution

The degree of substitution (DS) of DGE was calculated based on ^31^P‐NMR quantification analysis using the following equation (2),

(2) $$\mathrm{DS}=C{}_{f}-C{}_{i}/C{}_{i}$$

where C_f_ and C_i_ are the final, and initial concentrations of hydroxy groups in the KL and DGE‐modified KL, respectively.

### Phenolic‐OH and Carboxylic‐OH Analysis

The amount of phenolic hydroxy and carboxylic‐OH groups in KL and MKL were analyzed by an automatic potentiometer (Metrohm, 728 Titrado, Switzerland), using the potentiometric titration method that was explained in the literature.^66^ In this set of experiments, 0.06 g of each sample was dissolved in 1 mL of 0.8 M potassium hydroxide in a 200 mL beaker, then 4 mL of 0.5 % para‐hydroxybenzoic acid was added as an internal standard. Then, 100 mL of deionized water was added to the mixture, and the mixture was titrated against 0.1 M HCl standard solution to determine the content of phenolic‐OH and carboxylic‐OH groups (mmol/g) following equations 3 and (3),

(3) $$\mathrm{Phenolic}\text{-}\mathrm{OH}\;\mathrm{group}\;\left(\mathrm{mmol}/g\right)=\;\frac{[(V_{2}`-\;V_{1}`)\;-\;(V_{2}-\;V_{1})]\times \;C\;}{m}$$

(4) $$\mathrm{Carboxylic}\text{-}\mathrm{OH}\;\mathrm{group}\;\left(\mathrm{mmol}/g\right)=\;\frac{[(V_{3}`-\;V_{2}`)\;-\;(V_{3}-\;V_{2})]\times \;C\;}{m}$$

Where C is the titrant concentration (i. e., 0.1 M HCl), and m is the dried mass of KL (g). V_1_, V_2,_ and V_3_ are the first, second and third endpoint volumes of HCl solution (mL) in the blank sample; while V_1_‘, V_2_‘, and V_3_‘ are the first, second and third endpoint volumes of HCl solution (mL) used for the actual samples, respectively.

### Elemental Analysis

The organic elements of KL and MKL derivatives were assessed using an elemental analyzer (Elementar, Vario Micro, Germans). In this set of experiments, 2 mg of KL, dried at 60 °C overnight, were analyzed for their carbon, hydrogen, nitrogen, and sulfur contents by following a previously established method.^67^, ^68^ An average of three independent experiments was reported in this work.

### Fourier Transform Infrared (FTIR) Spectroscopy

To analyse the chemical structure of unmodified and modified KL, 50 mg of each sample was dried overnight at 60 °C to remove any moisture. Then, FTIR spectra were recorded using a Bruker Tensor 37, Germany, with ATR accessory. The spectra were recorded in the transmittance mode in the range of 500 cm^−1^ to 4000 cm^−1^ with resolution 1 cm^−1^.^65^ Each sample was scanned multiple times for consistency.

### Molecular Weight Analysis

KL and MKL derivatives were not water‐soluble. Therefore, their molecular weight determination was only possible using a static light scattering (SLS) technique. Each sample was prepared at five different concentrations (0.2. 0.4, 0.6, 0.8, and 1 g/L) in DMF solution and stirred at 500 rpm overnight. These solutions were then filtered with 30 mm nylon syringe filters having a 0.45 μm pore size (Celltreat Scientific Products). The intensities of the scattered light were measured using a static light scattering instrument (Brookhaven BI‐200SM, Holtsville, NY), which was attached to a goniometer at various angles between 15° and 155° while the laser wavelength was set at 637 nm, with narrow bandpass filter (center wavelength (CWL) tolerance of±2 nm) built into the detector. Finally, the generated data was analyzed using BIC Zimm Plot software.^51^, ^69^

### Thermal Analysis (TGA and DSC)

To determine the thermal stability of KL, 8–10 mg samples were dried for two days at 60 °C to remove any moisture. Then, each sample was loaded in a platinum crucible, and thermogravimetric analysis of the samples were performed using a TGA instrument, Instrument Specialist Inc i‐1000 series. Samples were heated isothermally at 100 °C to ensure the removal of any moisture. Then, they were heated from room temperature to 700 °C at 10 °C/min heating rates under nitrogen (35 mL/ min).^70^

The thermal behavior of KL derivatives was investigated with a differential scanning calorimeter (TA instrument, Q2000 DSC) using the standard cell RC mode. All samples were dried overnight at 60 °C before the DSC analysis. Approximately, 10 mg of each sample was loaded into a hermetic aluminum pan and analyzed in the heat/cool/heat mode between 30 and 250 °C at a rate of 5 °C/min under 50 mL/min nitrogen flow. In the second heating cycle, the glass transition and melting temperatures of the samples were determined.^42^

## Conflict of interest

The authors declare no conflict of interest.

## Supporting information

As a service to our authors and readers, this journal provides supporting information supplied by the authors. Such materials are peer reviewed and may be re‐organized for online delivery, but are not copy‐edited or typeset. Technical support issues arising from supporting information (other than missing files) should be addressed to the authors.

Supplementary
