# Supplementary material for: Modification of Kraft Lignin with Dodecyl Glycidyl Ether
Source: ChemistryOpen. 2019 Oct 10;8(10):1258–66. doi: 10.1002/open.201900263 (PMC6786095; doi:10.1002/open.201900263)
Supplement: Supplementary file 1 — Supplementary [file OPEN-8-1258-s001.pdf]

Open Access
ChemistryOPEN

Supporting Information

© Copyright Wiley-VCH Verlag GmbH & Co. KGaA, 69451 Weinheim, 2019

Modification of Kraft Lignin with Dodecyl Glycidyl Ether

Norah S. Alwadani and Pedram Fatehi*© 2019 The Authors. Published by Wiley-VCH Verlag GmbH & Co. KGaA. This is an open access article under the terms of the Creative Commons Attribution License, which permits use, distribution and reproduction in any medium, provided the original work is properly cited.

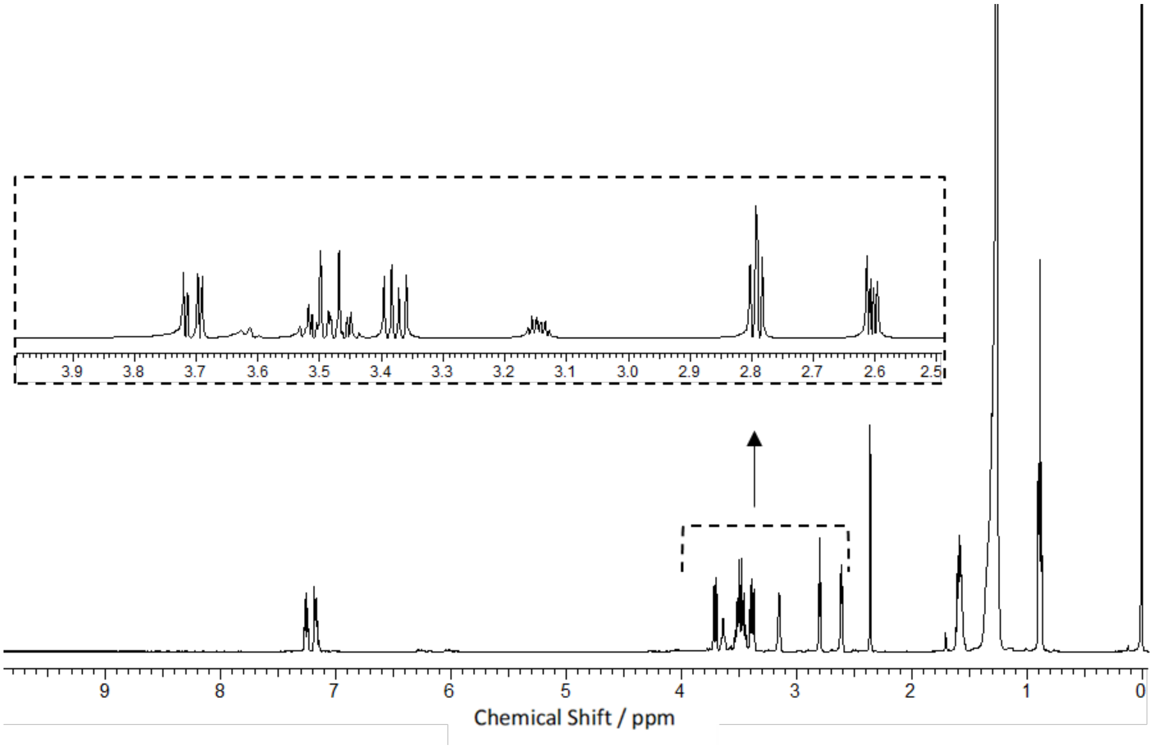

Figure S1: ^{1}H NMR spectrum of the DGE in $CDCl_3$.

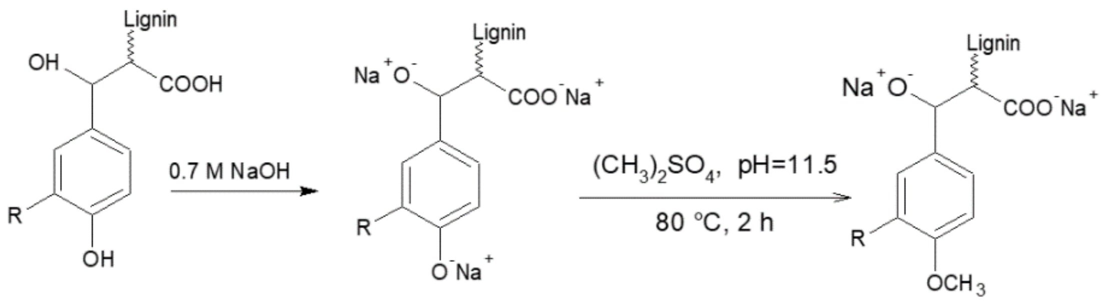

Scheme S1: The reaction route for methylation of kraft lignin.

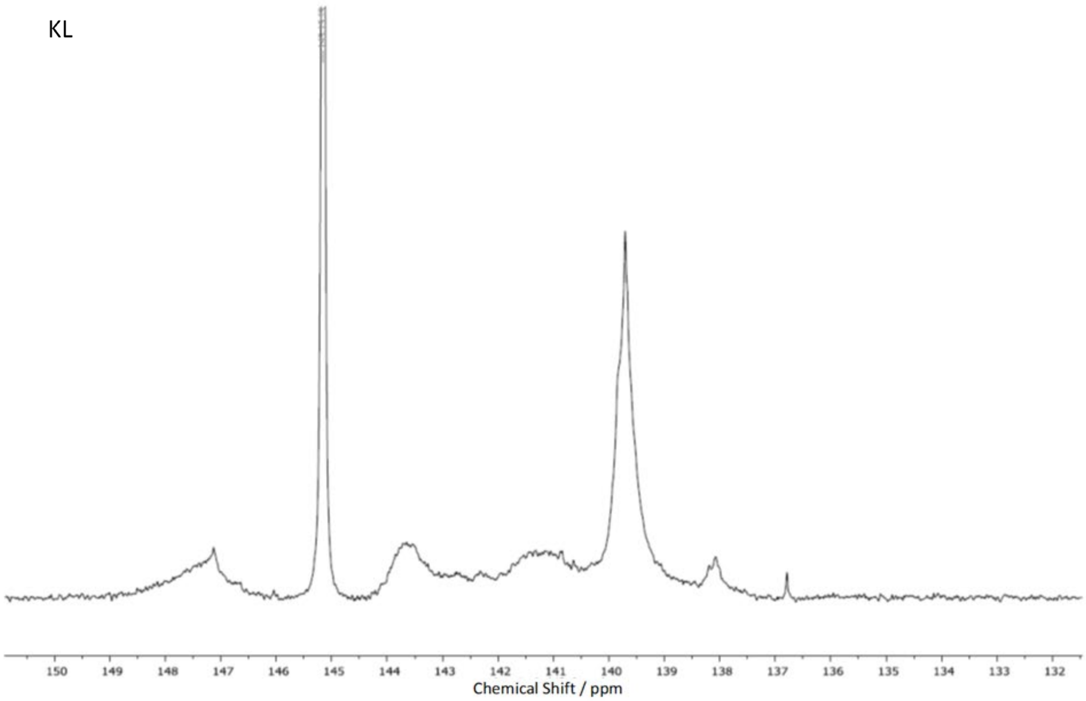

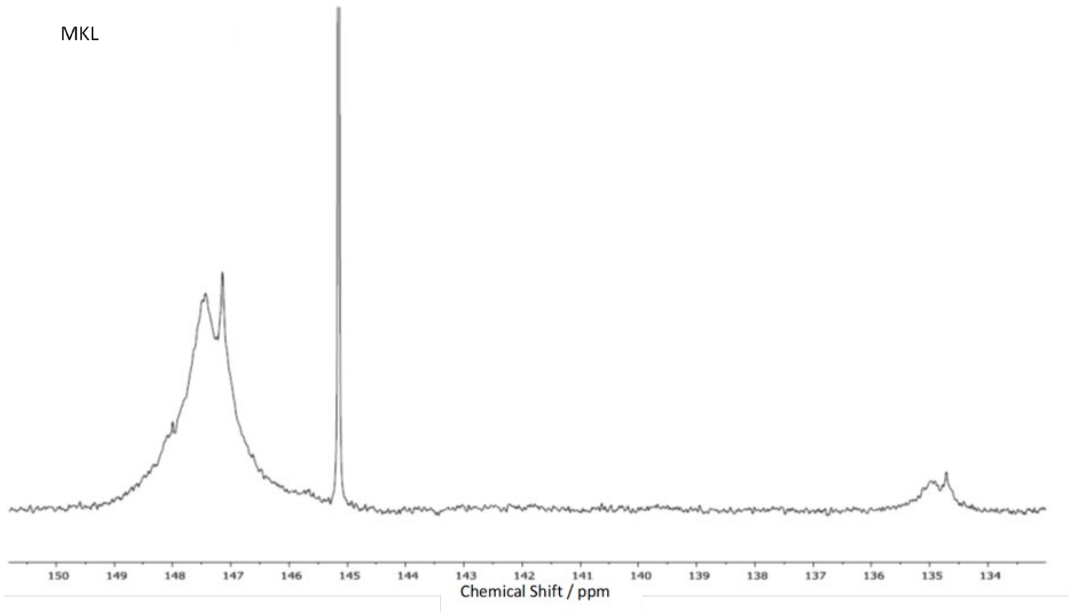

Figure S2: Quantitative ^{31}PNMR spectrum of kraft lignin (KL) and methylated kraft lignin (MKL) in pyridine/$CDCl_3$ mixture (1.6/1 v/v).

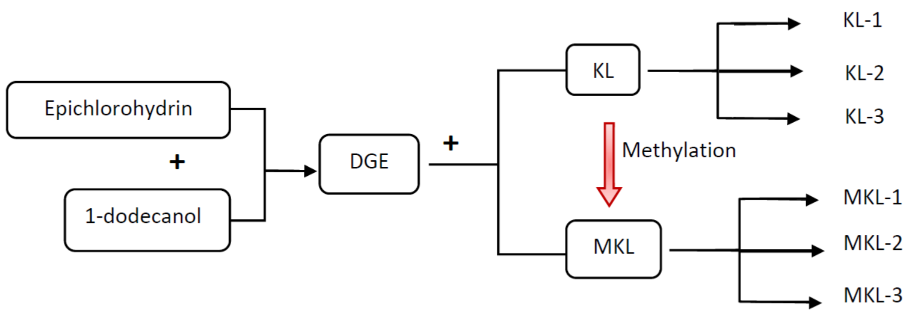

Figure S3: Overall reaction routes for producing DGE-grafted KL and DGE-grafted MKL.
